# Combination of Interventions Needed to Improve Maternal Healthcare Utilization: A Multinomial Analysis of the Inequity in Place of Childbirth in Afghanistan

**DOI:** 10.3389/fgwh.2020.571055

**Published:** 2020-12-08

**Authors:** Christine Kim, Daniel Erim, Kayhan Natiq, Ahmad Shah Salehi, Wu Zeng

**Affiliations:** ^1^Department of Health Policy and Management, Gillings School of Global Public Health, University of North Carolina at Chapel Hill, Chapel Hill, NC, United States; ^2^Health Economics and Outcomes Research (HEOR) Modeling and Advanced Analytics, Parexel International, Durham, NC, United States; ^3^Silk Route Training and Research Organization, Kabul, Afghanistan; ^4^Department of Global Health Development, London School of Hygiene and Tropical Medicine, London, United Kingdom; ^5^Department of International Health, School of Nursing & Health Studies, Georgetown University, Washington, DC, United States

**Keywords:** maternal health care utilization, place of childbirth, Afghanistan, health equity, healthcare access

## Abstract

Giving birth with a skilled birth attendant at a facility that provides emergency obstetric care services has better outcomes, but many women do not have access to these services in low- and middle-income countries. Individual, household, and societal factors influence women's decisions about place of birth. Factors influencing birthplace preference by type of provider and level of public facility are not well understood. Applying the Andersen Behavioral Model of healthcare services use, we explored the association between characteristics of women and their choice of childbirth location using a multinomial logistic regression, and conducted a scenario analysis to predict changes in the childbirth location by imposing various interventions. Most women gave birth at home (68.1%), while 15.1% gave birth at a public clinic, 12.1% at a public hospital, and 4.7% at a private facility. Women with higher levels of education, from households in the upper two wealth quintiles, and who had any antenatal care were more likely to give birth in public or private facilities than at home. A combination of multisector interventions had the strongest signals from the model for increasing the predicted probability of in-facility childbirths. This study enhances our understanding of factors associated with the use of public facilities and the private sector for childbirth in Afghanistan. Policymakers and healthcare providers should seek to improve equity in the delivery of health services. This study highlights the need for decisionmakers to consider a combination of multisector efforts (e.g., health, education, and social protection), to increase equitable use of maternal healthcare services.

## Introduction

Despite a relative decline of 43.9% in global maternal mortality from 1990 to 2015, more progress is needed to meet the United Nations Sustainable Development Goal (SDG) 3.1—a global maternal mortality ratio (MMR) of fewer than 70 deaths/100,000 live births by 2030 (1). Notably, most deaths and childbirth complications occur in low- and middle-income countries (LMIC) from preventable causes (2, 3). Several interventions have been introduced to reduce maternal mortality, including increasing skilled birth attendants (SBA) for delivery and access to in-facility births (4).

Recommendations pertaining to place of delivery include encouraging low-risk births at home with a SBA to all births in facilities where access to emergency obstetric care is available (5, 6). A third option leaves place of birth to patient and provider preference (5, 6). These preferences may be limited by barriers to accessing facilities as well as individual, household, and societal factors, e.g., needing permission from her husband, inaccessible facilities to accompanying relatives, perceived quality of care, previous experience with provider, cost, transportation, and distance (7–12). Globally, in-facility births ranged from below 40% among the poorest countries to above 90% among middle-income countries (6). Ninety-eight percent of in-facility births were performed by a SBA and 96% of SBA deliveries were in a facility, indicating that, globally, few home births and not all in-facility births were assisted by a SBA (6). This is particularly true for fragile and conflict-affected countries like Afghanistan, among the 11 countries with the lowest in-facility births (<40%); however, <10% of home births were with a SBA (6).

Afghanistan has among the highest maternal death rates in the world, nearly ten times greater than the global MMR (1, 13). While national MMR estimates are debated, regardless of the estimate or data source used, maternal deaths in the country are unacceptably high (14). Despite over 20 years of continuous conflict, significant progress has been made to rebuild the country's health system (15). Between 2003 and 2015, there were increases in the number of births with a SBA (14–51%) and in a facility (13–48%) (15, 16). However, current coverage estimates fall short of the recommended 87% SBA and 81% in-facility births required to achieve SDG 3.1 (1).

Socioeconomic and demographic factors, such as wealth quintile, parity, and education are strong predictors of SBA use in Afghanistan (17–21). Additionally, use of antenatal care (ANC) reduces delays in seeking care for obstetric emergencies (22). Despite an increase in ANC use in Afghanistan, over half of women give birth at home, with only 43% of childbirths in a public facility, and 5% in a private facility (16). Prior studies have shown utilization preferences for health services. For instance, one found that receiving health services in a public or private facility varied by type of service; public facilities were used more for inpatient services and less for outpatient services (23). Public health services in Afghanistan were designed for rapid scale-up through contracting with non-governmental organizations (NGOs) (24). This delivery approach improved the equity of health services provision by reaching the poorest households via NGO implementing partners, where the government lacked capacity to delivery services itself (25). However, as in other LMICs, there is an inequitable use of public compared to private facilities among the poorest and wealthiest households, respectively (23, 26).

Understanding the factors associated with women's place of birth preference is important for decisionmakers to improve planning and administration of maternal health services, such as through health education outreach programs, improved access to emergency obstetric care services, or training of health workers to improve quality of ANC. Unfortunately, factors influencing birthplace preference by type of provider (public vs. private) and level of public facility (clinic vs. hospital) are not well understood in Afghanistan.

We adapted the Andersen Behavioral Model (27) to understand the maternal healthcare utilization context. For this study, this model conceptualizes maternal healthcare use as a function of predisposing (socio-demographic factors), enabling (income and vehicle access), and need (woman's view of her own health or symptoms) factors. Feedback loops show that maternal health status influences predisposing, enabling, and perceived need for services, as well as behaviors (health practices during pregnancy and in-facility childbirth), which subsequently affect maternal health outcomes. Using the Anderson Behavioral Model, we explore the associations between key socio-economic and demographic factors and the likelihood of giving birth in a public clinic, public hospital, private facility, or at home (28).

## Materials and Methods

### Data Sources

**Figure 1:**
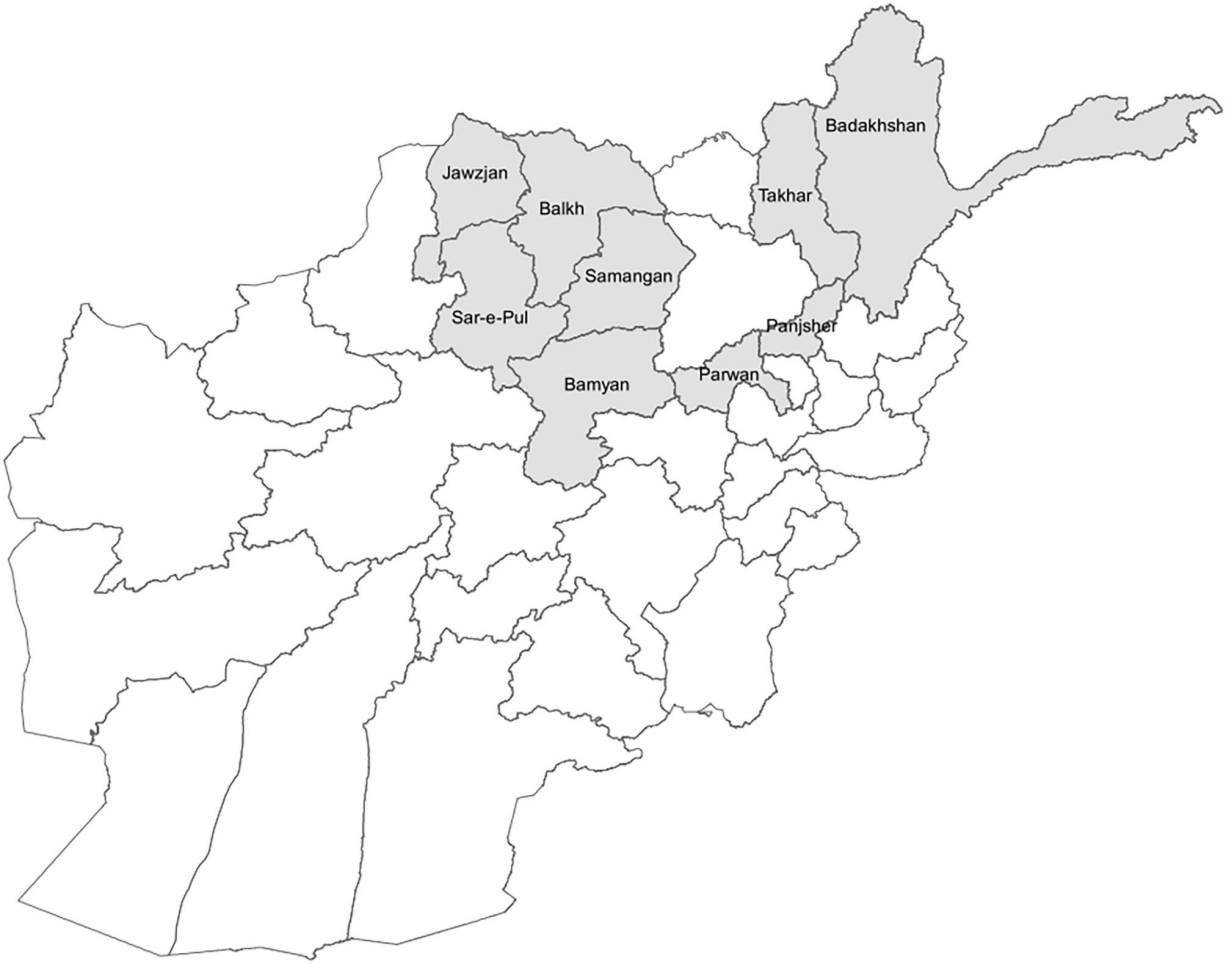
Provinces included in household survey for results-based financing pilot intervention.

We used household survey data which was conducted as part of an impact evaluation of a results-based financing (RBF) intervention in nine provinces in Afghanistan (Figure 1) (29, 30). The surveys are repeated cross-sections from 2010, 2013, and 2015. Two questionnaires were used for the household survey: the household questionnaire and the female and child health questionnaire.

The household survey used a multi-stage probability sample of the nine selected provinces purposively selected based on representation by different donors and feasibility of implementing the pilot project. In the first stage, 74 health facilities were randomly selected, stratified and matched by facility type and number of outpatient visits in the previous year. The second stage consisted of randomly selecting 288 villages or clusters in the catchment area of the selected health facilities (two clusters per health facility). Using the household listing conducted prior to the survey, the third stage involved simple random sampling of households in the selected villages. The same villages and health facilities were visited during each year of the survey. However, households may or may not be the same as they were not linked across the datasets. On average, interviews were conducted in 24 households per village, resulting in total household sample sizes of 6,878 in 2010, 6,848 in 2012, and 6,584 in 2015. There was less than a 1% nonresponse rate by households across all 3 years, and <5% missing data across variables.

The household questionnaire collected information from the head of the household on all household members, including name, sex, education, socioeconomic status, and utilization of health services. The female and child health questionnaire collected information from ever-married women age 15–49 or women over 18 years who are primary caretakers of children under 5 years of age; questions included age; literacy; pregnancy-based reproductive history; knowledge and use of family planning methods; access, utilization, and quality of services for antenatal care, delivery, postnatal care, and child health services, including immunization; and, perception and quality of services and trust in healthcare providers.

Data on insecurity incidents by province were obtained from the International NGO Safety Organization (31). We also used spatial data of villages included in the study sample and the country's road network. Spatial data were obtained from several publicly available sources (2012–2013 Afghanistan Information Management Services (AIMS) through the Humanitarian Data Exchange) and the MoPH (32).

### Measures

**Figure 2:**
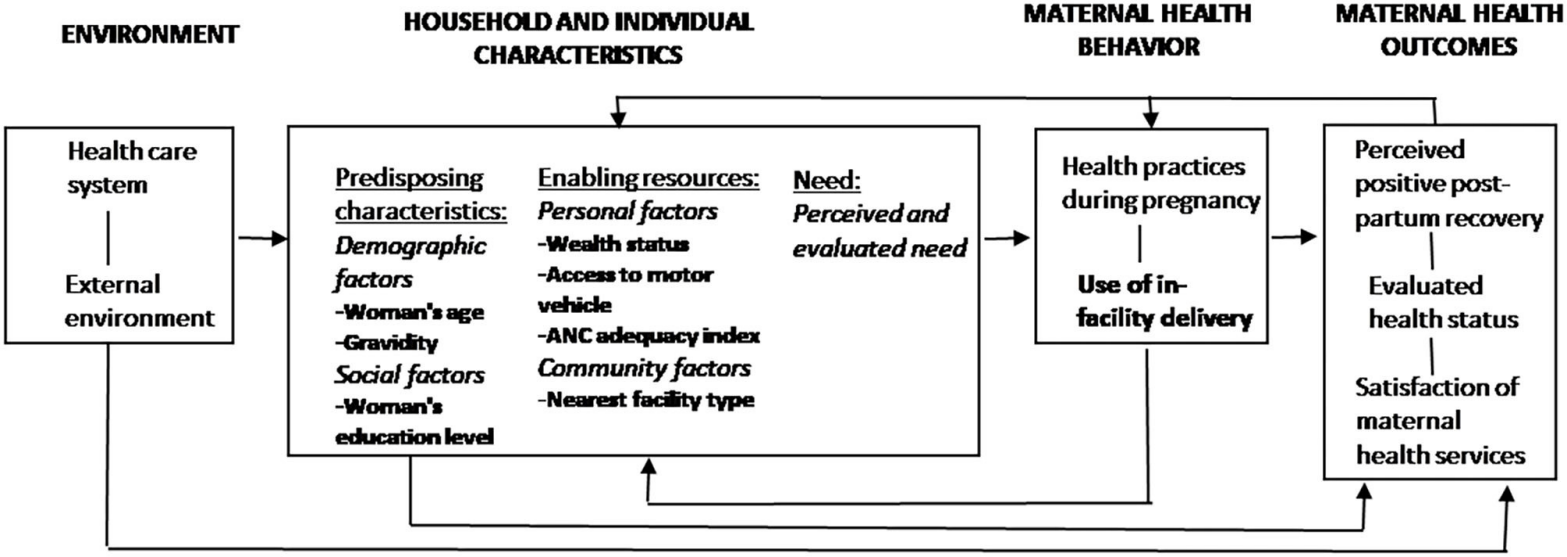
Conceptual model of maternal health care utilization adapted from Andersen's Behavioral Model. Variables included in the analysis are in bold.

The outcome variable was the location of childbirth with four options: public clinic, public hospital, private clinic or hospital, and home. Specifically, public clinics include the primary care clinics providing the Basic Package of Health Services (Comprehensive Health Centers, Basic Health Centers, and Sub Health Centers). Public hospitals include District Hospitals, Provincial Hospitals, and Regional Hospitals. Private clinic or hospital category includes any childbirth delivery in the private sector. Only 2% of home births were attended by a SBA, so Home includes all home births, the majority of which did not have a SBA. Women with an in-facility birth all reported having a SBA. Individual and household-level determinants of health access were chosen based on the Andersen Behavioral Model (Figure 2) (28). The predisposing factors in our model include the woman's age (divided into seven five-year categories), education level (no education, primary, secondary or higher), and gravidity (no previous pregnancies or >1 previous pregnancies). Enabling factors include household wealth status [quintile constructed based on index scores (33)], distance to the nearest main or secondary road (in km) as a proxy for rurality, access to a motor vehicle for transport (yes or no), number of security incidents in the province of residence (continuous), provincial health performance score (continuous), type of nearest public health facility (Hospital, Comprehensive Health Center, Basic Health Center, or Sub-Health Center), and an ANC adequacy index (no ANC, inadequate, and adequate). We calculated the Euclidean distance to the nearest main or secondary road (in km) using the country's road network and geocoded locations of villages included in the study sample in ArcGIS.

Some studies have used ANC as a proxy for need by assuming that women who use ANC are likely to have knowledge of the services or perceive the need due to problems during pregnancy (34, 35). However, receiving ANC is likely to lead to childbirth in certain locations because the woman is better connected to the health care system and not necessarily because she has more need. Thus, ANC adequacy index was included as an enabling factor.

The ANC adequacy index was based on four dimensions of continuity and adequacy: skill of provider (physician, nurse, or midwife), timeliness (care initiated during the first 4 months of pregnancy), sufficiency (at least four ANC visits during the pregnancy), and appropriateness (summary indicator of nine services received: weight check, blood pressure check, urine exam, blood test, nutrition counseling, counseling on signs of pregnancy complications, counseling on where to go when complications occur, iron and folic acid supplementation, and tetanus toxoid 2 vaccine). Studies in LMICs commonly use the receipt of >4 ANC visits due to limited data availability. We modified the Adequacy of Prenatal Care Utilization Index to generate this ANC adequacy index to account for timeliness, content, and process of care based on WHO recommendations as the number of visits alone do not correlate with content and process of care received (36, 37). Use of an ANC index is limited in the LMIC context, however, the need to standardize and better measure effective ANC coverage has been recognized (37, 38). We used a threshold of six services received, relaxing the 80% threshold used in similar studies for “good” or “adequate” ANC that used medical record data (39, 40). ANC was considered adequate if a woman had at least four skilled ANC visits in the first 4 months of pregnancy with at least six of the nine services received. ANC was considered inadequate if a woman had at least one skilled ANC visit after the first 4 months of pregnancy and less than six services received.

Covariates in the model include the province of residence, whether the woman lived in the catchment area of a facility that received the RBF intervention, and year (2010, 2013, 2015).

### Statistical Data Analysis

We accounted for the survey design and weights at each level. A multinomial logistic regression was used to assess the relationship between the characteristics of women and their choice of childbirth location; because giving birth at home has the highest likelihood of maternal-related complications and death and considered undesirable location from a policy perspective, it was selected as the comparison category. We assumed complete case analysis given the low level of missingness across the variables (<5%).

Descriptive statistics were weighted to account for the survey design. Multicollinearity was assessed by obtaining the variance inflation factors (VIF); security incidents and provincial health performance score were both excluded from the final model due to their high levels of correlation with the province variable. Results from the multinomial logistic regression analyses are presented as average marginal effects (AME) with 95% confidence intervals (CI). All statistical analyses were done in Stata 14 (StataCorp).

### Scenario Analysis

We used the multinomial logistic model to predict changes in the location of childbirth by altering variables that showed significance in the model as well as our conceptual framework. To provide greater context on the associations identified through the model, we assessed potential policy-relevant variables as single scenarios and a combination of scenarios, but differences were not tested for statistical significance. We assumed full coverage of each scenario in the sample. Based on the model results and our conceptual model, we examined changes in predisposing (education level) and enabling (motor vehicle access, ANC adequacy) factors. We used the method of recycled predictions in which the variables of interest varied across the entire sample, on average, while holding other variables constant (41). The results of individual scenarios and a combination of scenarios are presented as predicted percentages (for interpretability) with 95% confidence intervals (Supplementary Table 1).

### Ethical Considerations

Permission to use all datasets for this secondary data analysis was granted by the Ministry of Public Health in Kabul, Afghanistan. We received an exemption from the Institutional Review Board of the authors' institute to conduct this study (#16-3202).

## Results

### Characteristics of Study Sample

Of 9,705 women in the study sample, 15.1% gave birth at a public clinic, 12.1% at a public hospital, 4.7% at a private facility, and 68.1% at home. Over half the women were between 20 and 29 years of age, and fewer than 10.0% received any formal education (Table 1). Among women who delivered at home, about 40.0% were from households in the lowest two wealth quintiles. Women who delivered at a facility, however, were overwhelmingly from households in the highest two wealth quintiles (45.0% of women at a public clinic, 60.0% at a public hospital, 70.0% at a private facility). Women who went to a public hospital or private clinic lived on average <10 km from the nearest main or secondary road; women who went to a public clinic or gave birth at home lived at least 20 km away. Among women who gave birth at home, about 47.0% did not receive ANC. On the other hand, about 75.0% of women who delivered in either a public clinic or hospital received inadequate ANC; only 10.0% received adequate ANC. Fewer women received adequate ANC (8.7%) among those who gave birth at a private facility.

**Table 1:** Characteristics of women included in study sample by delivery location used for most recent birth within the last 2 years, accounting for complex survey design^*^ (*n* = 9,705).

	**Total**	**Public clinic**	**Public hospital**	**Private clinic/ hospital**	**Home**	
	**(*n* = 9,705)**	**(*n* = 1,468)**	**(*n* = 1,176)**	**(*n* = 455)**	**(*n* = 6,606)**	
	***n* (%)**	***n* (%)**	***n* (%)**	***n* (%)**	***n* (%)**	***p*-value**
**PREDISPOSING FACTORS**						
**Woman's age category**						
15–19 years	575 (5.9)	106 (7.7)	88 (8.3)	31 (5.8)	350 (5.0)	≤ 0.001
20–24 years	2,545 (26.3)	443 (30.6)	343 (28.8)	136 (30.2)	1,623 (24.5)	
25–29 years	2,576 (26.2)	361 (24.6)	289 (22.9)	107 (23.4)	1,819 (27.3)	
30–34 years	1,778 (18.2)	240 (15.3)	199 (18.2)	69 (14.4)	1270 (19.1)	
35–39 years	1,406 (13)	219 (14.9)	158 (13.4)	70 (16.1)	959 (15.2)	
40–44 years	632 (6.6)	80 (5.7)	75 (6.3)	31 (7.1)	446 (6.8)	
45–49 years	191 (2.0)	19 (1.1)	23 (2.2)	11 (3.0)	138 (2.1)	
**Woman's education level**						
No education	8,877 (91.5)	1,297 (88.1)	985 (84.5)	368 (81.9)	6,227 (94.3)	≤ 0.001
Primary education	415 (4.3)	92 (6.5)	77 (6.4)	32 (7.1)	214 (3.1)	
Secondary education or more	410 (4.2)	79 (5.4)	113 (9.1)	55 (9)	163 (2.6)	
**Gravidity**						
No previous pregnancies	264 (2.9)	39 (2.4)	39 (3.3)	7 (1.5)	179 (3.1)	0.147
1 + previous pregnancy	9,439 (97.1)	1,429 (97.6)	1,137 (96.7)	448 (98.5)	6,425 (96.9)	
**ENABLING FACTORS**						
**Wealth status**						
First quintile	1,676 (16.8)	202 (14.6)	115 (11.0)	31 (7.0)	1,328 (19.0)	≤ 0.001
Second quintile	1,834 (18.9)	294 (20.5)	136 (12.0)	44 (7.0)	1,360 (20.6)	
Third quintile	1,948 (20.4)	279 (19.0)	187 (16.2)	66 (13.4)	1,416 (22.1)	
Fourth quintile	2,007 (21.2)	310 (20.9)	278 (25.1)	101 (23.4)	1,318 (20.4)	
Fifth quintile	2,240 (22.7)	383 (23)	460 (35.7)	213 (47.6)	1,184 (18.0)	
**Access to motor vehicle**						
No	8,625 (93.6)	1,312 (93.3)	1,031 (89.5)	402 (89.3)	5,880 (94.8)	≤ 0.001
Yes	593 (6.4)	105 (6.7)	125 (10.5)	49 (10.7)	314 (5.2)	
**Nearest facility type**						
Sub Health Center	4,045 (45.0)	669 (45.4)	477 (44.3)	245 (59.2)	2,654 (43.9)	≤ 0.001
Basic Health Center	3,343 (28.0)	464 (28.8)	359 (23.6)	96 (14.3)	2,424 (29.6)	
Comprehensive Health Center	1,675 (20.2)	263 (21.3)	184 (18.0)	68 (16.0)	1,160 (20.6)	
Hospital	642 (6.9)	72 (4.5)	156 (14.2)	46 (10.0)	368 (5.9)	
Distance to the nearest main or secondary road (km)	9,406 (18.4)	1,402 (21.5)	1,150 (8.9)	447 (8.3)	6,407 (20.1)	0.056
Number of security incidents (mean)	9,705 (14.9)	1,468 (13.0)	1,176 (16.8)	455 (26.6)	6,606 (14.1)	≤ 0.001
Provincial health performance score (mean)	9,705 (59.9)	1,468 (59.3)	1,176 (60.2)	455 (59.3)	6,606 (60.1)	≤ 0.001
**ANC adequacy index (at least 4 visits)**						
No ANC	3,720 (36.3)	221 (14.1)	179 (15.2)	71 (14.1)	3,249 (47.0)	≤ 0.001
Inadequate	5,460 (58.3)	1,095 (75.6)	870 (74.6)	349 (77.2)	3,146 (49.8)	
Adequate	525 (5.4)	152 (10.3)	127 (10.2)	35 (8.7)	211 (3.2)	
**OTHER CONTROL VARIABLES**						
**Province**						
Parwan	1,610 (13.2)	204 (11.0)	333 (19.9)	82 (13.3)	991 (12.5)	≤ 0.001
Badakhshan	784 (7.8)	37 (2.2)	90 (7.6)	10 (2.2)	647 (9.6)	
Takhar	1,610 (16.6)	192 (13.2)	105 (9.2)	53 (11.7)	1,260 (19.1)	
Samangan	891 (10.7)	233 (18.9)	59 (5.8)	17 (3.8)	582 (10.2)	
Balkh	1,511 (14.8)	126 (7.3)	199 (18.7)	102 (19.2)	1,084 (15.5)	
Jawzjan	704 (10.7)	111 (10.6)	106 (13.5)	148 (40.9)	339 (7.9)	
Bamyan	1,479 (14.8)	332 (19.5)	175 (14.5)	14 (3.0)	958 (14.6)	
Saripul	535 (7.4)	110 (10.4)	49 (7.2)	12 (3.6)	364 (7.1)	
Panjsher	581 (4.0)	123 (6.9)	60 (3.6)	17 (2.3)	381 (3.6)	
**Year**						
2010	3,957 (40.1)	430 (29.6)	393 (33.2)	127 (25.0)	3,007 (45.0)	≤ 0.001
2013	3,222 (33.0)	478 (32.0)	322 (26.7)	183 (39.3)	2,239 (33.9)	
2015	2,526 (26.9)	560 (38.4)	461 (40.1)	145 (35.6)	1,360 (21.1)	
**Received RBF intervention**						
No	4,817 (51.1)	744 (54.0)	483 (42.1)	177 (40.7)	3,413 (52.8)	0.013
Yes	4,888 (48.9)	724 (46.0)	693 (57.9)	278 (59.3)	3,193 (47.2)	

*ANC, antenatal care; RBF, Results-based financing*.

* *Complex survey design, weights, and nested structure accounted for in descriptive analysis. Sub-total sample sizes may not be consistent due to missingness*.

### Relationship Between Women's Characteristics and Place of Birth: Multivariate Results

Table 2 presents the results from the multivariate multinomial logistic regression with home births as the base outcome. Education was significantly associated with a woman giving birth at a public clinic, public hospital, or a private facility compared to at home. Women with primary education had a 4.3% point increase in the predicted probability of childbirth at a public clinic [95% CI (0.0, 8.5)]. The average marginal effects of primary and secondary education were a 4.5 and 6.7% point increase in the predicted probability of childbirth at a public hospital [95% CI (1.3, 7.7); (2.9, 10.5)]. Women with secondary education had a 4.0% point increase in the predicted probability of childbirth at a private facility compared to home [95% CI (1.4, 6.5)]. Wealth status was significantly associated with a woman giving birth at a public clinic or private facility compared to home. Being in the second, fourth, and fifth quintiles compared to the first quintile was associated with a 4.1, 3.3, and 4.7% point increase in the predicted probability of childbirth at a public clinic, respectively [95% CI (1.5, 6.7); (0.2, 6.4); (1.1, 8.3)]. Being in the fifth wealth quintile compared to the first quintile was associated 3.6% point increase in the predicted probability of childbirth at a private facility [95% CI (1.0, 6.2)]. ANC adequacy index was significantly associated with a woman giving birth at any facility option compared to home. Receipt of inadequate ANC compared to no ANC was associated with a 13.5, 8.5, and 3.0% point increase in the predicted probability of childbirth at a public clinic, public hospital, or private facility, respectively [95% CI (11.3, 15.7); (6.7, 10.4); (1.9, 4.0)]. Larger associations were found between in-facility births and the receipt of adequate ANC compared to no ANC {22.6, 12.9, and 3.8% point increase in the predicted probability of childbirth at a public clinic, public hospital, or private facility, respectively [95% CI (17.4, 27.8); (9.3, 16.6); (1.4,6.3)]}.

**Table 2:** Multinomial regression analysis of women's characteristics and childbirth location for most recent birth within the last 2 years (*n* = 8,921).

	**Place of birth (reference: Home birth)**		
	**Public clinic**	**Public hospital**	**Private clinic/hospital**
**Variables (reference group)**	**AME**	**AME**	**AME**
	**(95% CI)**	**(95% CI)**	**(95% CI)**
**PREDISPOSING FACTORS**			
**Woman's age category (ref: 15–19 years)**			
20–24 years	−0.024 (−0.068, 0.021)	−0.032 (−0.072, 0.008)	0.011 (−0.008, 0.031)
25–29 years	−0.056^*^ (−0.099, −0.012)	−0.055^**^ (−0.096, −0.014)	−0.001 (−0.021, 0.018)
30–34 years	−0.069^***^ (−0.111, −0.027)	−0.040^*^ (−0.080, 0.000)	−0.007 (−0.027, 0.014)
35–39 years	−0.044 (−0.090, 0.001)	−0.047^*^ (−0.090, −0.004)	0.006 (−0.015, 0.027)
40–44 years	−0.068^**^ (−0.119, −0.018)	−0.034 (−0.080, 0.012)	0.008 (−0.016, 0.033)
45–49 years	−0.119^***^ (−0.177, −0.062)	−0.021 (−0.084, 0.041)	0.041 (−0.006, 0.088)
**Woman's education level (ref: No education)**			
Primary education	0.043^*^ (0.000, 0.085)	0.045^**^ (0.013, 0.077)	0.021 (−0.002, 0.044)
Secondary education and more	0.016 (−0.019, 0.052)	0.067^***^ (0.029, 0.105)	0.040^**^ (0.014, 0.065)
**Gravidity (ref: No previous pregnancies)**			
1+ previous pregnancies	0.021 (−0.023, 0.065)	−0.020 (−0.063, 0.024)	0.028^**^ (0.009, 0.047)
**ENABLING FACTORS**			
**Wealth status (ref: First quintile)**			
Second quintile	0.041^**^ (0.015, 0.067)	−0.024 (−0.054, 0.006)	−0.015 (−0.033, 0.003)
Third quintile	0.020 (−0.011, 0.051)	−0.004 (−0.036, 0.028)	0.006 (−0.016, 0.028)
Fourth quintile	0.033^*^ (0.002, 0.064)	0.027 (−0.008, 0.061)	0.017 (−0.004, 0.038)
Fifth quintile	0.047^*^ (0.011, 0.083)	0.030 (−0.005, 0.065)	0.036^**^ (0.010, 0.062)
**Main mode of transportation (ref: Foot)**			
Motor	−0.006 (−0.043, 0.031)	0.022 (−0.005, 0.049)	0.005 (−0.013, 0.023)
**Closest facility type (ref: Sub Health Center)**			
Basic Health Center	0.019 (−0.015, 0.053)	−0.016 (−0.049, 0.017)	0.012 (−0.007, 0.031)
Comprehensive Health Center	0.005 (−0.029, 0.039)	−0.032 (−0.067, 0.004)	−0.005 (−0.024, 0.014)
Hospital	−0.002 (−0.069, 0.066)	0.060^*^ (0.005, 0.115)	0.001 (−0.023, 0.025)
Distance to the nearest main or secondary road (km)	0.001 (0.000, 0.001)	−0.003^***^ (−0.003, −0.002)	0.000 (−0.001, 0.000)
**ANC adequacy index (ref: No ANC)**			
Inadequate	0.135^***^ (0.113, 0.157)	0.085^***^ (0.067, 0.104)	0.030^***^ (0.019, 0.040)
Adequate	0.226^***^ (0.174, 0.278)	0.129^***^ (0.093, 0.166)	0.038^**^ (0.014, 0.063)
**OTHER CONTROL VARIABLES**			
**Province (ref: Parwan)**			
Badakhshan	−0.067^***^ (−0.106, −0.028)	−0.012 (−0.074, 0.051)	−0.019^*^ (−0.036, −0.002)
Takhar	0.040 (−0.026, 0.107)	−0.084^***^ (−0.122, −0.046)	0.007 (−0.012, 0.026)
Samangan	0.196^***^ (0.136, 0.256)	−0.028 (−0.080, 0.023)	−0.003 (−0.024, 0.018)
Balkh	−0.043^*^ (−0.078, −0.008)	−0.014 (−0.068, 0.040)	0.035^**^ (0.011, 0.060)
Jawzjan	0.010 (−0.031, 0.050)	−0.025 (−0.065, 0.015)	0.121^***^ (0.069, 0.173)
Bamyan	0.081^**^ (0.031, 0.130)	−0.015 (−0.071, 0.040)	−0.028^***^ (−0.042, −0.014)
Saripul	0.064^*^ (0.001, 0.127)	0.017 (−0.040, 0.075)	0.001 (−0.026, 0.028)
Panjsher	0.151^***^ (0.080, 0.222)	−0.063^***^ (−0.101, −0.025)	−0.013 (−0.032, 0.005)
**Year (ref: 2010)**			
2013	0.034 (0.011, 0.058)	−0.012 (−0.031, 0.007)	0.034^***^ (0.018, 0.049)
2015	0.085^***^ (0.057, 0.113)	0.056^***^ (0.031, 0.082)	0.031^***^ (0.013, 0.048)
**Received RBF intervention (ref: No)**			
Yes	−0.016 (−0.045, 0.014)	0.022 (−0.003, 0.046)	0.016^*^ (0.001, 0.032)

*AME, Average marginal effect; ANC, Antenatal Care; RBF, Results-based financing*.

* *p ≤ 0.05*,

** *p ≤ 0.01*,

*** *p ≤ 0.001. Marginal effect for factor variables is the discrete change from the base level*.

### Scenarios of Utilization Patterns of In-Facility Births

**Figure 3:**
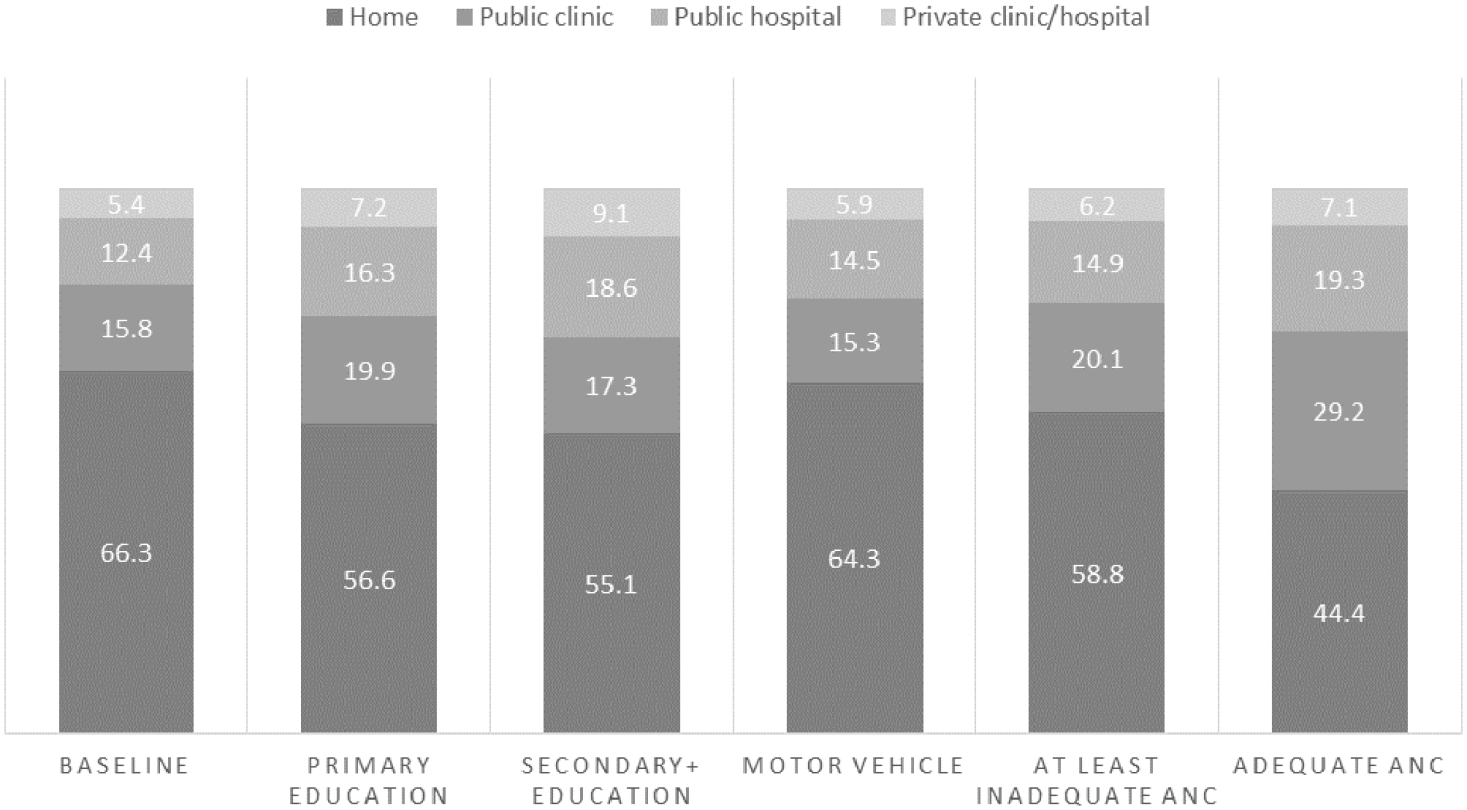
Predicted percentages of childbirth location given single variable scenarios.

**Figure 4:**
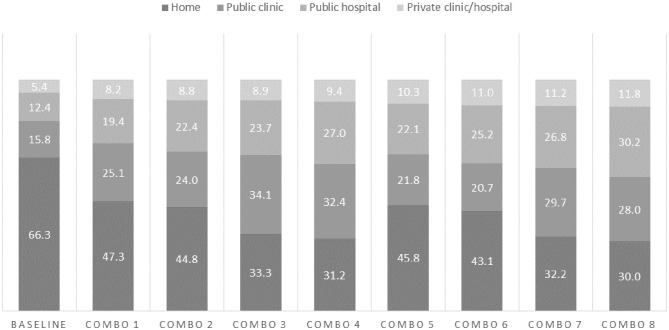
Predicted percentages of childbirth location given a combination of scenarios*. *Combo 1: Primary education + at least inadequate ANC; Combo2: Primary education + at least inadequate ANC + motor vehicle; Combo 3: Primary education + adequate ANC; Combo 4: Primary education + adequate ANC + motor vehicle; Combo 5: Secondary education + at least inadequate ANC; Combo 6: Secondary education + at least inadequate ANC + motor vehicle; Combo 7: Secondary education + adequate ANC; Combo 8: Secondary education + adequate ANC + motor vehicle.

Figures 3, 4 present the results of the various scenarios of target variables and their predicted changes on utilization patterns of in-facility births at public clinics, public hospitals, private facilities, and home, assuming all individuals reach the full potential of the target variables. Overall, adequate ANC as a single variable yielded the largest changes in decreased home childbirth (from 66.3 to 44.4%). However, a combination of variables was the most effective in decreasing home childbirths. Increased access to secondary education and adequate ANC was associated with a decrease in home births from 66.3 to 32.2%, with 29.7% at public clinics, 26.8% at public hospitals, and 11.2% at private facilities. The addition of a motor vehicle to this combination predicted further decreased home births (to 30.0%). Increased access to primary education, adequate ANC, and motor vehicle decreased home births to 31.2% with 32.4% at public clinics, 27.0% at public hospitals, and 9.4% at private facilities. Despite assessing the full coverage of the combined scenarios, about 30.0% of women were predicted to still give birth at home.

## Discussion

Using the Andersen Behavioral Model, the findings show that women with higher levels of education, greater household wealth, and having any ANC were more likely to give birth in public or private facilities than at home. The scenario analysis found that a combination of variables, particularly access to secondary education, adequate ANC, and motor vehicle for care, would have the largest change on the predicted decrease of home births and increase of in-facility births, particularly at public clinics and hospitals. These results are consistent with the literature and highlight the need for decisionmakers to consider a combination of multisector efforts (e.g., health, education, and social protection), to increase equitable use of maternal health services.

We found that primary and secondary education increased a woman's predicted probability of giving birth at a higher-level facility. Maternal education has been found to be a key determinant of maternal health utilization (42, 43). Women with higher levels of education often know when to seek care, understand benefits of preventive care, and are more empowered to actually seek care (44). Education can serve as a tool to enhance a woman's confidence and ability to make decisions for herself and seek out higher quality care (45, 46). However, within the context of strongly patriarchal societies, studies have shown the value of engaging husbands and other household decision makers, such as mothers-in-law, in educational sessions for improving maternal and child health (10, 43, 47). The ability of a woman to seek maternal health care is correlated with her household decision-making status and level of her husband's education, occupation, and support (48–50). Further, a woman's ability to obtain secondary education has been a consistent predictor of maternal health service utilization (51, 52). These factors should not be overlooked when designing programs to either jointly engage husbands and mothers-in-law or to recognize their role as a precondition to successful maternal health education interventions, as well as education for young girls.

Similar to other studies (11, 23, 45), we found that the poorest women were more likely to deliver at home and wealthier women were more likely to deliver in the private sector. We also found that women in the second poorest wealth quintile were more likely to use a public clinic for childbirth compared to the poorest women. Public clinics, which deliver the BPHS, have been found to be more equitable than other facility types (public hospitals or private facilities) in Afghanistan (23, 25). However, the poorest women may still face barriers to accessing the lowest levels of the health system for maternal healthcare. Although public clinics should be free of charge, patients often make informal payments for services and must bear the additional costs for such things as transportation and food, likely unaffordable for the poorest women (10, 20). High out-of-pocket payments by households remains a major barrier to accessing healthcare in Afghanistan (53). Households reported spending on transportation for healthcare services as the second highest expense following medicines and supplies (54). Policies are needed to address financial barriers to accessing maternal healthcare services, particularly for transportation, such as through vouchers, cash transfers, or community transport options to cover the cost and availability of transportation to a health facility.

Use of ANC was positively associated with giving birth at any of the facility types. Receiving adequate ANC had almost twice the effect as inadequate ANC in the likelihood of childbirth at a public clinic. The timing and number of ANC visits have been associated with a woman's preference on where to give birth (55, 56). Inadequate services during ANC can influence a woman's decision to seek additional care during pregnancy or childbirth. ANC visits are the best opportunity to support women in birth preparedness and to understand signs of pregnancy complications and how to seek emergency obstetric care when needed. Birth preparedness has been positively associated with in-facility births and as a means to reduce maternal and newborn deaths (57–59). Implementation of birth plans during ANC visits should be considered as a strategy to improve the uptake of maternal health services, including the number and content of ANC visits, skilled attendant at birth, and post-natal care. However, observed associations between ANC use and in-facility delivery may be suspect of resulting from confounding factors such as availability of and access to services, knowledge of pregnancy risks, and attitudes toward health services (60).

Although motor vehicle access was not significant in the model, it is commonly cited as a barrier to accessing facilities in qualitative studies and in other studies. Adding this variable to the scenario analysis achieved the highest predicted increase of in-facility births. Where resources are limited and increasing the number of facilities infeasible, countries like Afghanistan may consider different modes of transport to influence access to needed health care [such as conditional cash transfer for transportation (61, 62)], particularly in rural areas with diverse terrain (63–65).

Increasing in-facility births requires ensuring quality of care and availability of services, personnel, and medicines. With increased demand for services, particularly in the public sector, decision makers should ensure continuity of maternal healthcare services from pregnancy to post-partum and increase the availability and quality of emergency obstetric care in public facilities. In-facility births alone may not decrease mortality (66) without ensuring sufficient quality of care (67, 68). In a five-country study in sub-Saharan Africa, the quality of basic maternal healthcare functions was substantially lower in primary care compared to secondary level facilities (67). There is a perception that private health facilities have better quality care, and evidence has shown that women who can afford to, prefer to bypass their nearest public clinic for a higher level clinic or hospital (20, 69). Equitable access to quality maternity healthcare should be considered, particularly as poorer women tend to utilize services at public clinics. Further, interventions targeting women who continue to deliver at home should increase access to birth planning support, knowledge of pregnancy complications and obstetric dangers signs, and access to SBAs.

We found that the combination of secondary education, adequate ANC, and motor vehicle in the scenario analysis had the strongest signals from the model for increasing the predicted probability of in-facility childbirths. The scenario analysis does not show causality between the multisector inputs and improved maternal health care utilization; however, it highlights the importance of multisector efforts to contribute to substantial changes in maternal healthcare utilization. Further, it highlights the need for health research to better measure and assess multisector factors on healthcare use and health-related outcomes. The SDGs comprise of 17 goals to spur action among countries to improve health, education, economic growth, and tackle climate change. SDG 4 specifically focuses on universal access to primary and secondary education which would help decrease maternal mortality as stated in SDG 3. Global efforts to build coherent policies through the SDGs need to similarly bridge interactions across sectors in countries for greater human development impacts (70). Achieving the SDGs requires multisectoral interventions that address poverty, food insecurity and malnutrition, universal health coverage, quality universal education, and environmental protection (71). Investments across the relevant sectors with policy interactions may not only decrease preventable deaths but also “ensure health and well-being, and expand enabling environments” (72). Multisector interventions should be studied to better understand their linkages to improving maternal health outcomes.

We note several limitations of this study. First, we were unable to identify or account for women with high-risk pregnancies. Failing to control for pregnancy risk may introduce confounding as it is likely to be on the causal pathway. Second, our study includes nine provinces in Afghanistan, thus limiting generalizability, particularly the southern provinces which are geographically and ethnically different from the central and northern regions and have greater insecurity. Studies have shown that maternal healthcare utilization and outcomes, as well as coverage of health facilities are poorer in the southern region (15, 16, 33, 73). Third, our sample was limited to women who delivered in the past 2 years so not all births were captured. However, this shortened recall period may reduce recall bias. Fourth, the situation in Afghanistan is volatile and the security levels change rapidly in the country. We were unable to control for district-level security incidents, and it is unclear how specific villages and households are affected. Yet, evidence has shown that conflict affects access and use of health services (74). Fifth, we did not account for clustering at the village level. The multinomial model would enhance the efficiency and is likely to reduce the standard errors of coefficients by dividing the total variance into variance at the group level and that at the individual level. Although our model did not account for the variation at the village level, with a lower efficiency of detecting potentially meaningful variables predicting the use of maternal services, the coefficients remained unbiased. Sixth, we did not have the full spectrum of women's preferences for place of childbirth usually determined by a discrete choice experiment, which was beyond the scope of this study. Finally, we did not have specific quality measures of care or perceptions of care that a woman received at her place of childbirth. Perception of quality and satisfaction of care may influence a woman's decision to seek care at a specific facility and should be accounted for in future studies.

This study enhances our understanding of factors associated with the use of public facilities and the private sector for childbirth in Afghanistan. Policymakers and healthcare providers should seek to improve equity in the delivery of health services, particularly for necessary emergency obstetric care. Strengthened collaborations between health and other sectors, such as increased access to education for girls, may address critical leverage points in improving maternal health outcomes and overall well-being. Further, there is a need to address the financial barriers to transportation to reach health facilities in a timely manner. Additional research is needed to investigate health system factors that are amenable to change such as health service quality to provide a more complete understanding of individual, health systems, and community level factors that influence a woman's place of childbirth.

## Data Availability Statement

The data analyzed in this study is subject to the following licenses/restrictions: Raw data were generated at the Silk Route Training and Research Organization and owned by the Afghanistan Ministry of Public Health. Derived data supporting the findings of this study are available from the corresponding author (CK) on request, with permission from the Afghanistan Ministry of Public Health. Several of the spatial datasets that support the findings of this study are openly available in the Humanitarian Data Exchange at data.humdata.org. Requests to access these datasets should be directed to Christine Kim, christine_kim@alumni.unc.edu;data.humdata.org.

## Ethics Statement

Permission to use all datasets for this secondary data analysis was granted by the Ministry of Public Health in Kabul, Afghanistan. We received an exemption from the Institutional Review Board of the authors' institute to conduct this study (#16-3202).

## Author Contributions

CK contributed to study conception and design, analyzed data, and drafted the manuscript. DE, KN, AS, and WZ contributed to critical review and manuscript revision. All authors contributed to the article and approved the submitted version.

## Conflict of Interest

DE was employed by the company Paraxel International. The remaining author declares that the research was conducted in the absence of any commercial or financial relationships that could be construed as a potential conflict of interest.

## Abbreviations

Abbreviations: ANC: Antenatal Care
BPHS: Basic Package of Health Services
LMIC: Low and middle-income country
MMR: Maternal mortality ratio
NGO: Non-governmental organization
RBF: Results based Financing
SBA: Skilled Birth Attendance
SDG: Sustainable Development Goals
VIF: Variance Inflation Factors.
