# Supplementary material for: Combination of Interventions Needed to Improve Maternal Healthcare Utilization: A Multinomial Analysis of the Inequity in Place of Childbirth in Afghanistan
Source: Front Glob Womens Health. 2020 Dec 8;1:571055. doi: 10.3389/fgwh.2020.571055 (PMC8594015; doi:10.3389/fgwh.2020.571055)
Supplement: Supplementary file 1 [file Table_1.DOCX]

Supplement Table 1 Predicted percentages of childbirth location given intervention scenarios

|  | **Public clinic** | **Public hospital** | **Private facility** | **Home** |
| --- | --- | --- | --- | --- |
|  | **%** | **%** | **%** | **%** |
|  | **(95% CI)** | **(95% CI)** | **(95% CI)** | **(95% CI)** |
| Baseline | 15.8 | 12.4 | 5.4 | 66.3 |
|  | (14.5, 17.2) | (11.2, 13.6) | (4.6, 6.2) | (64.7, 67.9) |
| *Single intervention scenarios* |  |  |  |  |
| Primary education | 19.9 | 16.3 | 7.2 | 56.6 |
|  | (15.5, 24.2) | (13.0, 19.7) | (5.0, 9.4) | (51.9, 61.2) |
| Secondary or more education | 17.3 | 18.6 | 9.1 | 55.1 |
|  | (13.8, 20.7) | (14.7, 22.4) | (6.8, 11.4) | (50.2, 60.0) |
| Motor vehicle | 15.3 | 14.5 | 5.9 | 64.3 |
|  | (11.5, 19.1) | (11.6, 17.3) | (4.3, 7.6) | (60.2, 68.4) |
| At least inadequate ANC | 20.1 | 14.9 | 6.2 | 58.8 |
|  | (18.1, 22.2) | (12.3, 16.5) | (5.3, 7.2) | (56.4, 61.1) |
| Adequate ANC | 29.2 | 19.3 | 7.1 | 44.4 |
|  | (24.2, 34.2) | (16.0, 22.6) | (4.8, 9.5) | (38.9, 49.8) |
| *Combination of interventions scenarios* |  |  |  |  |
| Primary education + at least inadequate ANC | 25.1 | 19.4 | 8.2 | 47.3 |
|  | (19.6, 30.6) | (15.4, 23.5) | (5.7, 10.7) | (41.6, 53.0) |
| Primary education + at least inadequate ANC + motor vehicle | 24.0 | 22.4 | 8.8 | 44.8 |
|  | (17.1, 30.9) | (16.9, 27.8) | (5.3, 12.3) | (37.8, 51.8) |
| Primary education + adequate ANC | 34.1 | 23.7 | 8.9 | 33.3 |
|  | (26.0, 42.2) | (18.2, 29.2) | (4.4, 13.3) | (26.8, 39.8) |
| Primary education + adequate ANC + motor vehicle | 32.4 | 27.0 | 9.4 | 31.2 |
|  | (23.8, 40.9) | (20.2, 33.8) | (4.3, 14.6) | (24.2, 38.2) |
| Secondary education + at least inadequate ANC | 21.8 | 22.1 | 10.3 | 45.8 |
|  | (17.3, 26.3) | (17.5, 26.6) | (7.7, 13.0) | (40.1, 51.5) |
| Secondary education + at least inadequate ANC + motor vehicle | 20.7 | 25.2 | 11.0 | 43.1 |
|  | (14.4, 27.0) | (19.2, 31.2) | (7.1, 15.0) | (35.9, 50.3) |
| Secondary education + adequate ANC | 29.7 | 26.8 | 11.2 | 32.2 |
|  | (23.1, 36.3) | (21.1, 32.6) | (6.5, 15.8) | (25.7, 38.8) |
| Secondary education + adequate ANC + motor vehicle | 28.0 | 30.2 | 11.8 | 30.0 |
|  | (20.4, 35.5) | (23.2, 37.4) | (6.4, 17.3) | (22.8, 37.2) |
